# The Value of Multimodality PET/CT Imaging in Detecting Prostate Cancer Biochemical Recurrence

**DOI:** 10.3389/fendo.2022.897513

**Published:** 2022-05-27

**Authors:** Jie Jiang, Xiaoxia Tang, Yongzhu Pu, Yong Yang, Conghui Yang, Fake Yang, Yadong Tian, Jindan Li, Hua Sun, Sheng Zhao, Long Chen

**Affiliations:** ^1^Department of PET/CT Center, Yunnan Cancer Hospital, The Third Affiliated Hospital of Kunming Medical University, Yunnan, China; ^2^Department of Pharmacy, The Second Affiliated Hospital of Kunming Medical University, Yunnan, China; ^3^Department of Urology, Yunnan Cancer Hospital, The Third Affiliated Hospital of Kunming Medical University, Yunnan, China

**Keywords:** prostate cancer, biochemical recurrence, PET/CT- Positron Emission Tomography Computed Tomography, imaging

## Abstract

Prostate cancer (PCa) induced death is the predominant cause of cancer-related death among men in 48 countries. After radical treatment, biochemical recurrence has become an important factor for prognosis. The early detection and diagnosis of recurrent lesions are very helpful in guiding treatment and improving the prognosis. PET/CT is a promising method for early detection of lesions in patients with biochemical recurrence of prostate cancer. This article reviews the progress of the research on PET/CT in the PCa biochemical recurrence and aims to introduce new technologies and provide more direction for future research.

## Background

Prostate cancer (PCa), the fifth reason of cancer-related death among male, is also the second most commonly diagnosed cancer (1). In 2020 1.4 million cases were newly diagnosed and 375,000 deaths were identified around the world (2). With the general promotion of prostate-specific antigen (PSA) screening, the improvement of biopsy technology, as well as the optimization of treatment methods, both of the incidence and mortality rates of PCa have declined or stabilized in most countries in recent years. However, the incidence of advanced PCa has increased (2, 3). Prostate cancer is a malignant tumor with extremely heterogeneous clinical behavior and has biological behaviors ranging from inertia and organ limitation to rapid invasion and easy metastasis (4). It is diagnosed mainly through digital rectal examination (DRE) and PSA testing. Once a preliminary diagnosis is made, a needle biopsy guided by a rectal ultrasound (TRUS) is performed (5). After an initial treatment *via* radical prostatectomy (RP) or local radiotherapy (RT), almost half of patients develop biochemical recurrence (BCR) and an increase in PSA. After a potential remedial treatment option, androgen deprivation therapy (ADT) is usually used for the patient. After the ADT, prostate-specific antigens begin to rise again in 2-8 years, and metastatic castration-resistant PCa can develop (6). Studies have shown that salvage RT (SRT) after early RP provides a cure for increased PSA in patients after RP (7–9), and therefore, early detection of BCR and lesion metastasis and accurate restaging guidance for the treatment of recurrent PCa is very important. Both of CT and MRI are structural imaging techniques and are of limited sensitivity and specificity for detecting a minimal metastatic lesion, which leads to a lower diagnostic rate for common imaging techniques in asymptomatic patients (10). The molecular imaging PET/CT is believed to be superior to BCR detection.

## PET/CT Imaging Agent for BCR PCa

BCR is generally defined by elevated PSA values (more than 0.2 ng/ml) in consecutively two tests after RP (11, 12). For patients receiving radiation therapy, biochemical failure is defined as the end of radiotherapy with the lowest PSA increase in the last 6 weeks being ≥ 2 ng/mL (13). Over the past decade, a variety of PET probes have achieved good results in detecting recurrent lesions and disease staging in PCa patients. PET radiotracers that are used have developed rapidly and mainly include radiolabeled choline, prostate specific membrane antigen (PSMA) ligands,^18^F-fluciclovine, gastrin-releasing peptide receptor(GRPR), fibroblast activation protein inhibitors(FAPI) and so on (14–22) (**Table 1**)

**Table 1:** Common PET-CT imaging agents to detect biochemical recurrence of prostate cancer.

Name	Half-life (min)	Production type	Mechanisms
^11^C-choline	20	Cyclotron	Cell membrane synthesis
^11^C-acetate	20	Cyclotron	Fatty acid metabolism
^68^Ga-PSMA-11	68	Generator	PSMA binding
^68^Ga-RM2	68	Generator	GRPR receptor binding
^68^ Ga-FAPI-04	68	Generator	FAP inhibitors
^18^F-FDHT	107	Cyclotron	Androgen receptor binding
^18^F-DCFPyL	107	Cyclotron	PSMA inhibitor
^18^F-NaF	107	Cyclotron	Bone chemisorption
^89^Zr-PSMA-DFO	4704	Generator	PSMA inhibitor

PET, positron emission tomography; ^18^F-FDG, ^18^F-fluoro-deoxy-glucose;

^18^F-NaF, sodium ^18^F-fluoride;GRPR,gastrin-releasing peptidere ceptor;

^18^F-FDHT,^18^F-fluorodehydrotestosterone;^18^F-DCFPyL,^18^F-2-(3-(1-carboxy-5-[(6-^18^F-fluoro-pyridine-3-carbonyl)-amino]-pentyl)-ureido)-pentanedioic acid; FAP,fibroblast activation protein.

**Figure 1:**
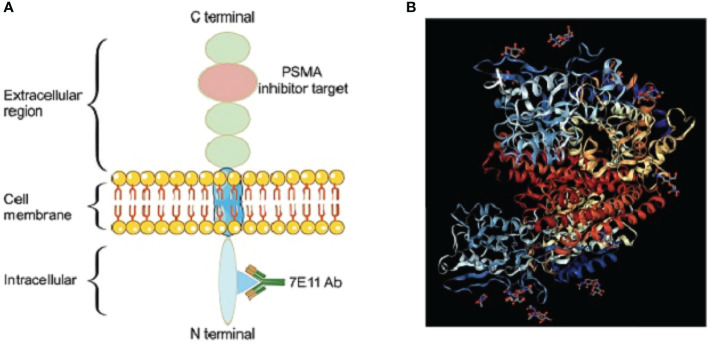
PSMA structure diagram. **(A)** Schematic illustration of PSMA. **(B)** Cyrstal structure of PSMA.

PSMA is a highly overexpressed transmembrane glycoprotein detected in the majority of prostate cancer cells (23) and is located in the apical region of prostate cells (i.e., the prostate tube) (**Figure 1**). PSMA is expressed in peripheral epithelial cells (24), and high-grade PCa have higher PSMA expression, and PSMA expression in late and castration-resistant PCa is further increased (25). PSMA binds with high affinity to the folate hydrolase of the PC cells, allowing the PSMA to show its potential to recognize BCR sites (26), and becomes the target of PCa imaging and therapy. PSMA-PET exhibits good early detection and localization of PCa recurrence lesions and identification of BCR lymph node metastasis after RP (27, 28). (**Table 2**) And ^68^Ga-PSMA-11 is the first FDA-approved radiotracer for PCa-specific PET/CT imaging (29, 30) (**Table 2**)

**Table 2:** The difference between PSMA and PSA.

	PSMA	PSA
Type	Integral cell-surface membrane protein	Secretory protein
Function	Several enzymatic functions	Known function-liquefaction of semen
Relationship with androgens	Upregulated with androgen deprivation	Decreased with androgen deprivation
monoclonal antibody	Ideal target for monoclonal antibody therapy	Not suitable for monoclonal antibody
Clinical Value	Neither related to clinical stage nor as diagnostic cancer marker	Useful in the clinical diagnosis and staging and an important diagnostic biomarker
Index meaning	The values may be controversial even if effective treatment	Fall to low values in response to effective treatment
Prognostic value	Elevated levels predict clinical progression or clinical resistance in more than 70% cases	Lower prognostic value

Choline radiopharmaceuticals were used for prostate cancer earlier, and often labeled with ^11^C and ^18^F, which can be used for the detection of recurrent lesions and the detection of early recurrence in patients with a history of BCR PCa (31, 32). ^18^F-fluciclovine is a synthetic amino acid with good biodistribution and little urinary disturbance, is often used for restaging of BCR patients (33). Gastrin-releasing peptide receptor antagonist (RM2) binds to GRPR on PCa cells, complementary to PSMA-targeted imaging (15, 34). Fibroblast activation protein (FAP) is highly expressed in a variety of epithelial cancers, and FAP inhibitor (FAPI) PET/CT has been used for various tumor imaging. Research has confirmed the uptake of ^68^Ga-FAPI-04 in PCa tissue was higher than that in normal prostate tissue, and FAP expression was increased after ADT, which has potential when the detection of lesions is limited after ADT (16, 21, 35).

## Recurrent Lesions Detection and Localization

Patients with a large number of RPs have an elevated PSA, and early detection and localization of anatomical sites of recurrence are critical to guide subsequent treatment. PET/CT was believed to be better than a morphological-based standard imaging mode (CWU) (36). An analysis of prostate cancer in Asian populations showed that standard imaging was not sensitive to recurrent PCa, and none of the bone lesions detected by PET was detected by CWU (37). Choline-PET is the most widely studied method, and although it has excellent specificity (38), its sensitivity is low, especially when PSA levels are low (39). A prospective study showed a PSMA-PET/CT detection rate of 66%, which is remarkably higher than the ^18^F-choline PET/CT detection rate of 32% (40). ^68^Ga-PSMA PET showed an obviously higher detection rate and a higher general impact on the clinical management than ^18^F-fluoromethylcholine (41, 42). ^18^F-fluciclovine has excellent detection rates for low, medium and high PSA levels (43, 44), and the test results are significantly better than those obtained with ^11^C-choline (38, 45). A meta-analysis showed that in BCR patients, the combined detection rates of ^18^F-labeled choline, fluciclovir, and PSMA were 66%, 74%, and 83%, respectively (38). The study by Hoffmann et al. compared the detection rates of ^18^F-PSMA and ^68^Ga-PSMA PET, and the results showed that the detection rates of the two tracers were similar, 87.5% (112/128) and 88.9% (121/136), respectively (46). A recent study showed, ^68^Ga-P16-093, a small molecule PSMA ligand, detected 71% of lesions in BCR patients (47) (**Table 3**)

**Table 3:** Detection rate of different imaging agents for BCR PCa.

Author	Year	Study type	Patients(n)	Imaging agents	Detection rate	Management change
De Man K (48)	2022	Prospective study	51	^18^F-PSMA-11	82%	52%
Abghari Gerst M (49)	2022	Prospective study	2005	^68^Ga-PSMA-11	78%	—
Ceci F (50)	2022	Retrospective study	189	^68^Ga-PSMA-11	54.5%	31%
Mena E (51)	2021	Retrospective study	245	^18^F-DCFPyL	79.2%	—
Zhou X (52)	2022	Retrospective study	71	^18^F-PSMA-1007	79%	—
Christensen MT (53)	2021	Retrospective study	532	^18^F-rhPSMA-7	80%	—
Lee H (47)	2022	Prospective study	15	^68^Ga-P16-093	71%	41%
Filippi L (54)	2022	Retrospective study	81	^18^F-fluciclovine	76.9%	31%
Zattoni F (55)	2021	Retrospective study	140	^18^F-Choline	70.7%	—
Wang R (38)	2021	Meta	5324	^18^F-choline	66%	—
			1706	^18^F-PSMA	83%	—
			1410	^18^F-fluciclovine	74%	—

Previous studies have shown that PSMA PET has a higher detection rate than other tracers, and some researchers have found that when PSMA expression is low or PSMA negative tumor area ≥ 50%, PSMA-PET results are negative, although PSA levels are very high (56). When PSMA expression is low, Dietlein et al. found 5 ^89^Zr-PSMA-DFO PET-positive lesions in 14 PSMA-PET-negative patients.^89^Zr-PSMA-DFO PET becomes a good supplement because its half-life is long enough to allow the process of ligand internalization to proceed sufficiently to make the lesions visible (22, 56, 57). Targeting gastrin-releasing peptide receptor (GRPR) is thought to complement PSMA-negative prostate cancer (PCa) patients (58), and it is helpful for the localization of recurrent lesions in ^18^FECH PET/CT-negative patients (59).Another study showed that the detection rate of ^18^F-FDG PET/CT in PSMA-PET negative patients was 16.7%, and patients with PSA ≥2.3 ng/mL and high Gleason score were more likely to benefit from FDG PET (60).

## Factors Affecting the Detection Rate

Many studies (41, 61–65) have indicated that PSMA, choline, fluciclovine PET/CT positive results possibly are significantly correlated with increased PSA levels (37). For patients with BCR, the positive rate of the PET/CT scan varies based on the clinical stage of the BCR, PSA levels as well as PSA doubling time during the scan are correlated with positive results (27, 66). A study showed that the detection rates of ^18^F-labeled choline, fluciclovine, and PSMA were 35, 23, and 58% for a PSA level less than 0.5 ng/ml;80, 92, and 94% for a PSA level more than 2.0 ng/ml (38). The rate of increase grows with a rise in the serum PSA levels before the PET (27, 67, 68). There are studies that shown that when the PSA levels higher than 0.2 ng/ml while the PSA velocity ≥ 1 ng/ml/year, there will be a positive PSMA scan (69), and with higher PSA levels, the PSMA-PET shows better diagnostic performance (28).

It has been documented that androgen deprivation therapy experience in BCR patients is correlated with the positive rate of PSMA-PET scans (69), and there is evidence that PSMA is induced with low doses of ADT at lower PSA levels (≤0.3 ng/mL). Imaging may enhance the positive scan rate (70), but further research is needed. In addition, the time to inject the imaging agent is related to the contrast of the image. For BCR with low PSA levels, imaging 3 hours after injection is more advantageous in terms of lesion contrast (71), which may also have an effect on the positive scan rate. Scanning technology and timing also have an impact on the positive rate. Morawitz et al. (72) found that ^68^Ga-PSMA-11 PET/CT scanning in the late abdominal and pelvic stage after emptying the bladder was helpful to detect missed local recurrence lesions. Uprimny et al. (73) improved the detection rate of lesions by using furosemide before scanning.

## PET Imaging for BCR of Low PSA Levels

**Figure 2:**
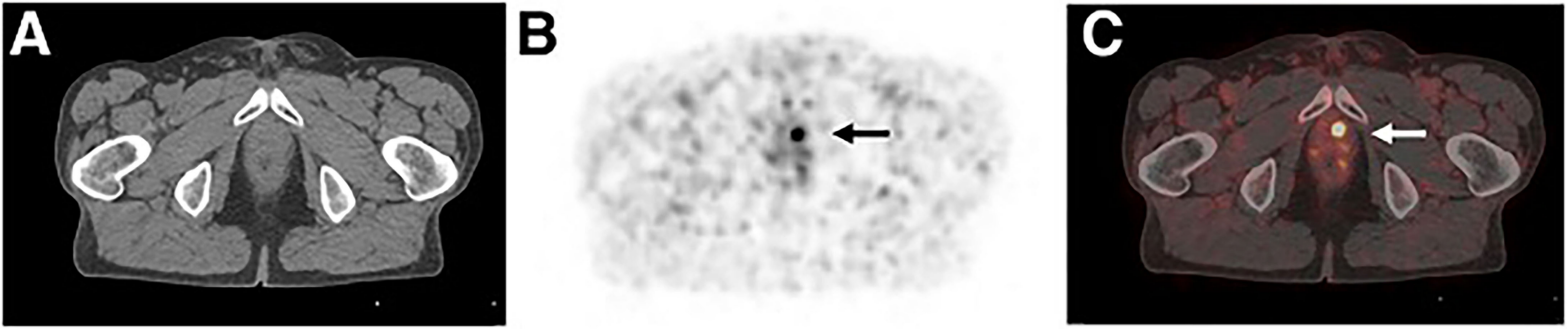
A 78-year-old patient with biochemical recurrence (PSA of 0.54 ng/mL) after radical prostatectomy (initially pT3b N0 M0 R0 G2). ^68^Ga-PSMA ligand PET/CT reveals focal uptake in left paramedian prostatic fossa, indicating local recurrence. The picture below shows transaxial CT **(A)**, PET **(B)**, and fused PET/CT **(C)** images respectively. Patient was referred for salvage radiation treatment. This research was originally published in JNM. Author(Schwarzenboeck SM, Rauscher I, Bluemel C, Fendler WP, Rowe SP, Pomper MG, Afshar-Oromieh A, Herrmann K, Eiber M). PSMA Ligands for PET Imaging of Prostate Cancer. J Nucl Med. 2017 Oct;58(10):1545-1552.^©^ SNMMI.

Currently, salvage RT (SRT) is one valuable treatments for patients with PSA elevation after RP. Early diagnosis of BCR at low PSA levels has a major impact on patients’ follow-up treatment. EVU guidelines recommend that PSA levels greater than 0.2 ng/mL and results influence subsequent treatment decisions, imaging of biochemically recurrent PCa with PSMA-labeled PET/CT (74). A study in 2005 patients with BCR found that the detection rate of ^68^Ga-PSMA-11 was 44.8% when the PSA was less than 0.25 ng/mL (49). A meta-analysis showed that the detection rates of ^18^F-Choline, ^18^F-Fluciclovine and ^18^F-PSMA PET/CT at PSA levels less than 0.5 ng/ml were 35%, 23%, and 58%, respectively (38). PEMA-PET is superior to other imaging methods at low PSA levels, as recommended by guidelines (**Figure 2**). ^18^F-fluciclovine PET is feasible for patients with PSA <1.0ng/ml. Filippi et al (54) found that the detection rate in 81 Italian patients was 66.7% when the PSA level was 0.2-0.57 ng/ml, and Wang et al. (75) in 46 patients with PSA level of 0.3-1.0ng/ml found the positive rate was about 33%, but it was not found positive cases in very low PSA (less than 0.3ng/ml) BCR patients. The value of ^18^F-fluciclovine PET in detecting lesions in BCR patients with very low PSA levels remains to be explored. A recent study found that at very low PSA (≤0.1ng/ml) levels, dynamic detection of ^11^C-choline PET was helpful in detecting early recurrence in BCR PCa patients (32). This could be a valuable new direction.

## Detection of Metastases in BCR Patients

**Figure 3:**
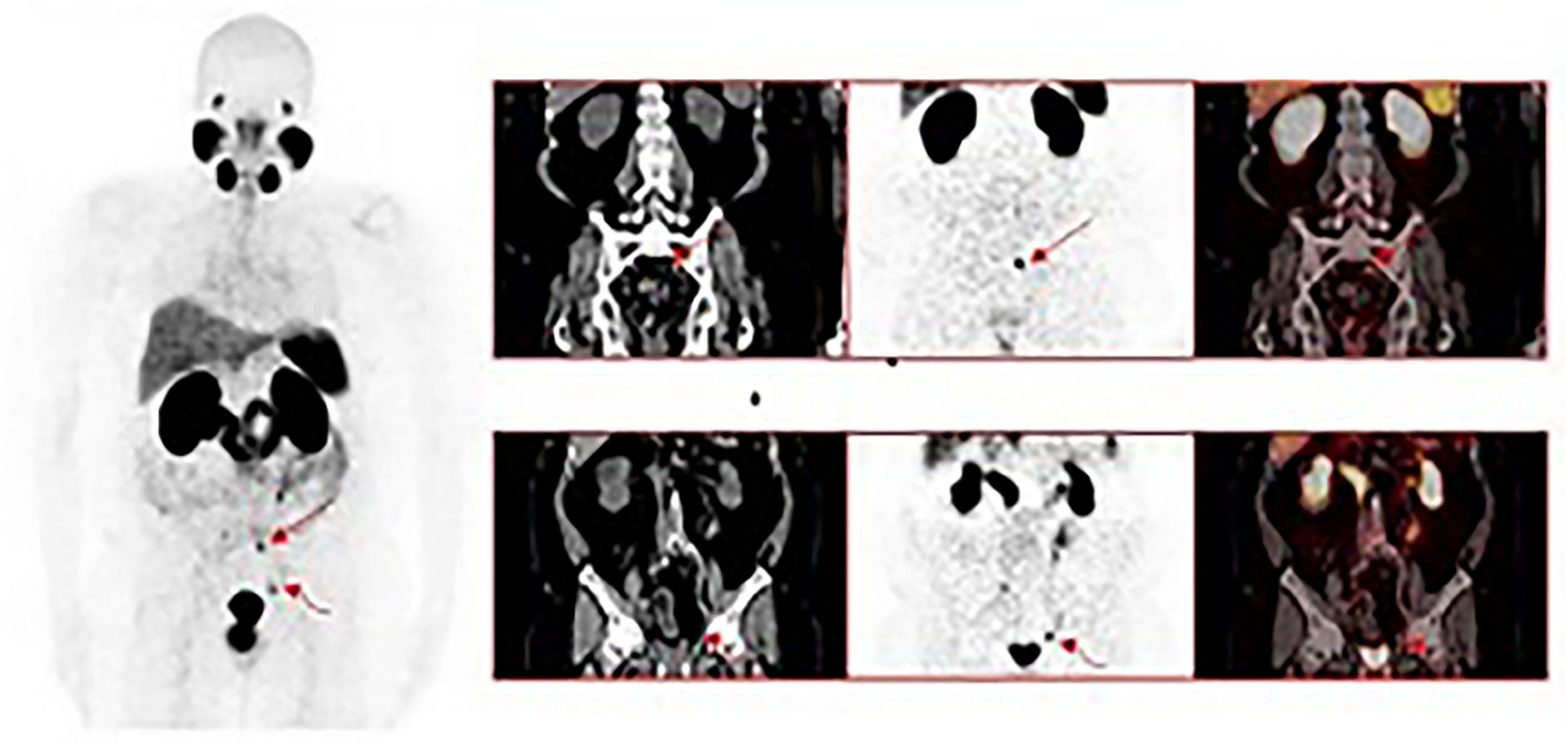
A 63-year-old male with a history of radical prostatectomy for adenocarcinoma of the prostate, Gleason 4 + 4. ^68^Ga-PSMA-11 PET/CT was requested for localization of disease recurrence at a serum PSA of 0.54 ng/mL. Images show intense tracer uptake in a subcentimeter left presacral node (straight arrows) and a subcentimeter left external iliac node consistent with the sites of prostate cancer recurrence. This research was originally published in JCM.Lawal IO, Lengana T, Popoola GO, Orunmuyi AT, Kgatle MM, Mokoala KMG, Sathekge MM. Pattern of Prostate Cancer Recurrence Assessed by 68Ga-PSMA-11 PET/CT in Men Treated with Primary Local Therapy. J Clin Med. 2021 Aug 29;10(17):3883.

Increased serum PSA levels are sensitive to *in vitro* markers of recurrent prostate cancer; however, it is still hard to differentiate local recurrence and regional or distant metastasis. Identifying metastatic disease can impact therapeutic schedule options and contributes to prognosis assessment (27). PSMA PET/CT is most commonly used to detect LN metastases and staging in BCR patients after RP, and its performance depends on the PSA levels as well as the volume of debris from metastatic cells (28). However, in small lymph nodes, this method performs well (76). Rauscher showed that ^68^Ga-PSMA PET detected LNM (77.9%) in 68 histopathologically confirmed metastatic LN regions, whereas conventional imaging modality only detected 18 of 67 regions (26.9%) (77). Studies have shown that ^18^F-rhPSMA-7 and ^18^F-rhPSMA-7.3 PET have a detection rate of 81.3% for lymph node metastasis in BCR patients after RP, and their accuracy in evaluating lymph node metastasis is comparable to that of ^18^F-PSMA-11 ^(^ 78).PSMA-PET/CT has higher diagnostic accuracy for lymph node recurrence after RP, especially for small-volume metastases, ^18^ F-PSMA-1007 PET/CT can reliably detect malignant lymph nodes larger than 3 mm with a specificity of over 99% (79–81) (**Figure 3**)

Bone metastasis is one of the common metastasis methods of prostate cancer, and it is difficult to differentiate diagnosis by traditional imaging due to degenerative changes. Mingels et al. found in 177 BCR patients that the PPV of ^18^F-PSMA-1007 PET to identify bone lesions was 79%, which was lower than the positive rate of the overall and LN (82). A meta-analysis showed that the highest sensitivity of NaF-PET/CT in identifying bone metastases was 0.97, followed by PSMA PET, higher than choline, MRI and bone scintigraphy (83). A recent study found that ^18^F-NaF PET/CT detected 93.6% of bone metastases, and the interobserver agreement was very high, with stable and reproducible results (18).

## PET/CT False Negatives and False Positives

For PSMA-PET, a small fraction (<10%) of PCa expressed low PSMA, which results in little or no uptake on PSMA-PET (79, 84). In these PSMA-negative patients, PSMA-PET is ineffective (36). In addition, metal artifacts low levels of PSMA uptake and bladder overflow are also possible elements of false negatives (85).Positive images need to be differentiated from normal tissue, benign lesions, and other non-PCa malignant lesions (25, 86–88). Reports have shown that in normal tissues, high or mild ^68^ Ga-PSMA-11 uptake was observed in the renal cortex, duodenum, parotid gland, and submandibular salivary glands, spleen, lacrimal gland, and liver (89–92). In some benign tissues with high proliferation rate, just like heart valves, pleura, endometrial scars, and granulation tissue, endothelial cells also express PSMA (25, 93, 94). Abnormal accumulation of PSMA-PET were detected in lots of benign lesions, including sarcoidosis (86, 95), Paget’s disease (96), healing fractures (97, 98), hemangioma (99), schwannomas (100), adenoma (101), and so on. Malignant tumors other than PCa, such as renal cell carcinoma (102, 103) and hepatocellular carcinoma (104), also have high expression. In addition, ^18^F-fluorocholine imaging usually shows abnormal uptake in lymph nodes due to inflammatory changes (105).All of the above cause false positive results, so it is necessary to learn about the physiological uptake and normal distribution in order to reduce the false positive results in the diagnosis.

## Conclusions

PSMA-PET has high accuracy in the detection of PCa BCR and the identification of metastasis, especially at low PSA levels. Its diagnostic potential is significantly better than that of choline and amino acid analogues, and this has a remarkable influence on managing patients in clinical. However, due to the expression and distribution characteristics of PSMA, it is unable to reliably recognize the PSMA-negative lesions, and other imaging methods need to be selected as supplements. According to the actual situation of patients, the combined use of complementary imaging agents to detect and locate BCR lesions is helpful for the early and effective detection and localization of recurrent lesions in BCR PCa patients, and is conducive to the selection of treatment options and the improvement of prognosis. Radiocomposites (such as ^18^F-NOTA-GRPR-PSMA, etc.) with the advantages of two or more imaging agents at the same time may become a research hotspot in the future.

## Author Contributions

HS conducted project management, writing review and editing. LC and SZ conducted method guidance, writing review and editing. JJ, XXT, YZP and YY conducted data collection, writing-draft preparation. CHY and FKY carried out illustration drawing and software support. YDT and JDL supervised the process. All authors contributed to manuscript revision, read, and approved the submitted version.

## Funding

This work was supported by the National Natural Science Foundation of China (grant number 81960496), Yunnan Fundamental Research Projects (grant number 202101AT070050), the Project funded by China Postdoctoral Science Foundation (grant number 2019M653501), Scientific Research Fund of Yunnan Province Educational Department (grant number K13219357), Yunnan Provincial Science and Technology Agency/Kunming Medical University Joint Project [grant number #2019FE001(-087)], Kunming Medical University Graduate Innovation Fund (grant number 2021S070), and the Xingdianyingcai support plan.

## Conflict of Interest

The authors declare that the research was conducted in the absence of any commercial or financial relationships that could be construed as a potential conflict of interest.

## Publisher’s Note

All claims expressed in this article are solely those of the authors and do not necessarily represent those of their affiliated organizations, or those of the publisher, the editors and the reviewers. Any product that may be evaluated in this article, or claim that may be made by its manufacturer, is not guaranteed or endorsed by the publisher.
